# Beyond conservative gender roles: exploring the division of paid and unpaid labour among Italian same-sex couples

**DOI:** 10.1186/s41118-025-00273-0

**Published:** 2025-11-07

**Authors:** Gioia Geremia, Agnese Vitali

**Affiliations:** 1 Centre d’Estudis Demogràfics (CED-CERCA), Universitat Autònoma de Barcelona, Barcelona, Spain; 2 Università degli Studi di Trento, Trento, Italy

**Keywords:** Same-sex couples, Division of labour, Gender norms, Doing gender, Specialisation

## Abstract

This contribution explores the division of paid and unpaid labour among same-sex couples in Italy relying on primary data collected via an online survey. The (non-probabilistic) sample consists of 190 respondents, mainly women (*n* = 138), in a co-residing same-sex couple at the survey date. Results from descriptive statistics reveal a general pattern of equal division of both paid and unpaid labour among the majority of couples in our sample—a result which aligns with previous research based on different countries. Same-sex partners in our sample tend to share domestic and childcare chores equally, even when paid labour is not equally shared. This result contrasts markedly with the gender division found among different-sex partners in Italy from existing empirical studies, especially among parents. Finally, we find that in those same-sex couples where the division of childcare is unbalanced, care tasks tend to be skewed towards the respondent irrespective of their relationship to the child, unlike prior international studies suggesting a higher involvement in care tasks for the birth or biological parent.

## Introduction

The emergence of new forms of partnerships and reproductive and family practices have challenged long-lived norms and expectations regarding gender roles within couples, especially in relation to parenthood. This shift is particularly relevant in the context of same-sex couples, whose growing diffusion has drawn increased scholarly attention (Boertien et al., 2024; Caprinali et al., 2023; Möllborn et al., 2025). However, these changes unfold within specific cultural and legal frameworks, as policies, laws and societal attitudes can significantly influence the experiences of same-sex couples, including the allocation of paid and unpaid labour among partners.

As of January 1st 2024, a total of 23,790 men and 13,965 women are in a same-sex civil union in Italy.^1^ An additional number of same-sex partners co-reside without having formalised their union. Despite the number of same-sex couples (SSC hereafter)in Italy has become non-negligible, little is known about their family dynamics and practices. In particular, the sociological and demographic literatures on the division of labour between partners in Italy has mainly focused on different-sex couples.

Empirical studies of the division of labour among SSC are predominantly based on the USA and Nordic European countries. Such literature has unequivocally found that both women and men in SSC tend to share domestic labour equally (Jaspers & Verbakel, 2013; Kurdek, 1993, 2007; Van der Vleuten et al., 2021). Furthermore, participation in the labour market is distributed more evenly among partners in SSC than in different-sex couples and the *breadwinner/homemaker* model is less frequent (Jaspers & Verbakel, 2013; Patterson et al., 2004). To explain these patterns, scholars argued that SSC are more likely to enact egalitarian practices since gender role constraints are less pressing compared to partners in different-sex couples (Peplau & Fingerhut, 2007). Childcare too tends to be equally shared between partners in SSC (Dunne, 2000; Tornello et al., 2015), although previous research suggests that birth mothers generally report greater involvement than social mothers (Downing & Goldberg, 2011; Goldeberg & Perry-Jenkins, 2007).

This paper has an exploratory aim—to describe how cohabiting SSC distribute paid and unpaid labour in Italy, a context where traditional gender norms remain pervasive, particularly in relation to parenthood. Heteronormativity is deeply rooted in the society and institutions, and the legal recognition of SSC only took place in 2016, with delay compared to other high-income countries. In such a gender-conservative context, partners in SSC may adopt an unequal division of labour, mimicking behaviours typical in different-sex couples (DSC hereafter), or they may prioritise equality between partners. These two scenarios might emerge either as a result of economic rationality, as suggested by the specialisation theory (Becker, 1985), or as a way of performing normative gender roles, as proposed by the doing gender approach (West & Zimmerman, 1987).

To explore the division of paid and unpaid labour among SSC in Italy, we rely on (non-representative) primary data collected through an online survey among individuals co-residing with a partner of their same sex. The survey collected information on household chores—whether the respondents or their partners perform a set of domestic tasks—and paid work—measured by the number of hours worked by each partner—alongside socio-economic and demographic information. Furthermore, the survey collected information on childcare, on whether the respondents or their partner was the biological parent of their child(ren) and, among women, whether the respondent or their partner was the birth mother. We use this information to describe whether there are unbalances towards the birth/biological or the social parent in the division of childcare.

In our sample, the majority of respondents in same-sex couples, both women and men, seem to equally divide housework and childcare. Such equal division is in place even when partners divide paid labour unequally, including when one partner is employed full-time and the other is not employed. Despite our sample is small and non-representative, results contributes to the debate about partnership dynamics among new family forms in a conservative setting.

## The Italian context

Gender culture plays a significant role in shaping the division of labour, in both different-sex (DSC) and same-sex (SSC) couples (Van der Vleuten et al., 2021). The perpetuation of conservative gender norms is embedded in the Italian familistic welfare regime (León & Migliavacca, 2013; León & Pavolini, 2017; Saraceno & Naldini, 2021). Women in DSC are consistently more involved in unpaid labour compared to men in Italy (Dotti-Sani, 2018a; García Román & Ophir, 2024). Time spent in housework and care work takes on 21.7% of the average day among women while only 7.6% among men.^2^

Italy lags behind in legal rights for same-sex couples. Civil unions among same-sex partners were legalised in 2016, considerably later than many other European countries, and only after the European Court of Human Rights ruled that Italy’s lack of legal recognition of same-sex families was a violation of the right to respect for family life. Marriage is unavailable to SSC and SSC are barred from accessing adoption and medically assisted reproduction. In 2024, gestational surrogacy was made illegal and prosecutable even if performed abroad in countries where it is permitted (Legislative decree no. 824/2024). Anti-LGBT hate crimes have increased in recent years (ILGA-Europe, 2024). Official statistics report that 61.2% of non-heterosexual employed people reported they hid their sexual orientation in the workplace, while 81.7% of gay and 78.8% of bisexual respondents experienced at least one form of micro-aggression in the workplace related to their sexual orientation (ISTAT-UNAR, 2021).

Yet, the share of young people who identifies as LGBTQ* is growing fast in Italy, reaching 11% and 23% of male and female (sex assigned at birth) university students, according to a sample collected among selected universities in 2023 (Castiglioni et al., 2025). The number of SSC also increased over time (Vitali et al., 2025). The growing prevalence of SSC in a still gender-conservative society might trigger opposite preferences concerning the division of labour among partners: the replication of heteronormative patterns, with specialisation of tasks among partners in order to maximise household utility, or defiance of conservative gender norms to challenge the conventional framework and equally share tasks between partners.

## Theoretical framework

### Between specialisation and doing gender: adapting existing theories to same-sex couples

Existing literature on the division of labour—both for the general population and for same-sex couples—has especially relied on two theories to explain the patterns found among couples: the *specialisation theory* (Becker, 1985), and the *doing gender* approach (West & Zimmerman, 1987).

*Specialisation theory* posits that DSC allocate paid and unpaid labour to maximise household utility, with each partner specialising in either paid or unpaid work based on their expected returns—considering income, education and occupational prestige (Becker, 1985). In DSC, this often aligns with women specialising in unpaid labour and men engaging in paid work (Craig, 2006). In SSC, however, this dynamic is less straightforward. Partners in SSC typically share similar socialisation experiences and do not face the physiological differences related to childbirth and breastfeeding that influence dynamics in DSC, since for women in SSC both partners have the potential to bear children and for men in SSC neither of the partners can (Evertsson et al., 2021).

The literature on DSC gives importance also to differences in relative resources—e.g., education—and to bargaining power, which partners use to strategically reduce their involvement in domestic labour (Lachance-Grzela & Bouchard, 2010). However, these seem to not affect the division of labour within SSC (Civettini, 2015; Shechory & Ziv, 2007; Solomon et al., 2005). Previous research also indicates that same-sex couples tend to exhibit greater educational homogamy than different-sex couples (Braack & Milewski, 2020). Empirical research indeed shows lower levels of specialisation among women (Aldén et al., 2015; Antecol & Steinberger, 2013; Evertsson & Boye, 2018; Perlesz et al., 2010) and men (Kurdek, 1993, 2007; Perlesz et al., 2010) in SSC compared to DSC. Not even the scarce educational homogamy typically associated with same-sex couples (Schwartz & Graf, 2009; Verbakel & Kalmijn, 2014) seem to affect the division of labour.

Differences in time availability can lead to partial specialisation, with the partner working longer hours in the labour market doing less housework (Aassve et al., 2014). Although presented as gender-neutral, this is a typical pattern found in DSC, where women spend less time in paid labour and invest more time in household tasks (Geist, 2005). This approach might easily translate to SSC, as shown by previous research (Civettini, 2015).

While specialisation theory (and time availability) might explain some patterns, it falls short of accounting for gendered variations within SSC—for instance, why women in SSC tend to equally share all tasks while men to specialise in specific tasks (Goldberg & Perry-Jenkins, 2007; van der Vleuten et al., 2021). The importance of gender in paid and unpaid labour stands out in the *doing gender* approach (West & Zimmerman, 1987), according to which gender is continuously constructed through everyday interactions with others: individuals may perform gender in ways that align with broader societal expectations. For instance, Carrington (2002) found that gay men tend to under-report the time spent on housework while lesbian women tend to over-report it. This might also apply to paid labour. Jaspers and Verbakel (2013) found that in the Netherlands women in lesbian couples were more likely to both work part-time, whereas men in gay couples were more likely to both work full-time.

Therefore, women in SSC may still feel compelled to engage more in domestic labour and have the same expectations for their partners, considering both socialisation and normative expectations about women and care (Blumstein & Schwartz, 1983; Kurdek, 2007). This might be particularly salient in conservative gender settings like Italy, where traditional gender norms are pronounced. At the same time, gender segregation and discrimination in the labour market, which often results in men occupying higher-paying positions, may indicate that men, given their higher economic resources, are able to more easily outsource (some) domestic tasks.

Given that, we might expect women in SSC to exhibit a more equal division of domestic labour than men. However, this does not necessarily imply differences between couple types in the *equality* of the division itself. For instance, women in SSC may perform more domestic labour overall—due to more outsourcing of tasks, for instance, among men. In this case, although men in SSC may spend less time on housework, the division between partners could still be relatively equal.

In summary, these frameworks suggest two competing scenarios. On one hand, SSC may resist heteronormative patterns and equally share paid and domestic labour. On the other, internalised gender norms and external pressures may reproduce traditional patterns. However, recent interpretations of the specialisation theory applied to SSC suggest that, when partners have similar socialisation experiences, specialisation is less likely since neither partner has a clear comparative advantage based on gendered expectations.

The intersection of these frameworks becomes particularly relevant when considering childcare. Additional mechanisms, not found in DSC, may be at play regarding the division of childcare among SSC with children. On one hand, physiological and institutional factors can reinforce traditional divisions even within SSC. For women in SSC with newborns (aged 0–2), the birth mother may take on more childcare due to childbirth and breastfeeding, thus possibly triggering (long-lasting) specialisation patterns among partners (Downing & Goldberg, 2011; Goldberg & Perry-Jenkins, 2007; Patterson, 1995). Moreover, birth mothers are entitled a period of compulsory (paid) maternity leave, which in many contexts is generally unavailable or considerably shorter for social mothers. Indeed, research on Swedish register data found that birth mothers in SSC take up more parental leave than social mothers (Evertsson & Boye, 2018; Moberg, 2016). In Italy, where parenthood for SSC is not legally recognised, the social mother is not entitled a period of paid parental leave, at least until stepchild adoption is formalized –generally several years after the birth of the child. As children grow, they start attending kindergarten,^3^ and the physiological impact of childbearing diminishes for the birth mother: that is why specialisation might lessen and a more equal division of childcare might emerge.

In contrast, men in SSC generally report a more equal distribution of childcare than women in SSC, as found by (scarce) previous studies (Tornello, 2015). This difference could partly stem from both the absence of physiological differences, and in the more challenging and costly access to parenthood that men in SSC have compared to women in SSC (e.g., in the Italian context gestational surrogacy is illegal and pursuing it abroad is costlier than accessing medically assisted reproduction for women in SSC). Men in SSC with children are likely a highly selected group, as accessing surrogacy requires significant financial resources. As a result, these couples might be more inclined to outsource domestic work—and potentially some childcare.

### Previous research in the USA and Europe

The literature on the division of labour in same-sex couples has been deeply influenced by previous research on different-sex couples. Among the latter, conservative gender roles and practices persist –despite reducing over time–, with women continuing to devote more time to domestic and care tasks and less time in paid work compared to men (Altintas & Sullivan, 2016; Pailhé et al., 2021).

Existing research on SSC mainly focused on the USA and, to a lesser extent, Europe. Both quantitative (Gotta et al., 2011; Kurdek, 2007; Matthews et al., 2002; Solomon et al., 2005) and qualitative studies (Esmail, 2010; Kelly & Hauck, 2015; Pfeffer, 2010) found that partners in SSC share paid and unpaid labour, especially domestic work, more similarly compared to DSC. While men in SSC tend to specialise in specific tasks, women in SSC share equally each and every task (Kurdek, 1993, 2007), including in Italy (Saraceno & Bertone, 2003).

An equal division of tasks among partners in SSC applies to childcare too (Chan et al., 1998; Patterson et al., 2004; Tornello, 2015). For men in SSC, allocation of paid and unpaid labour seems to be unaffected by parenthood (Sutphin, 2013; Tornello, 2015). However, among women in SSC, patterns of specialisation emerge in some US studies, where the birth mother is more involved in childcare compared to the social mother (Downing & Goldberg, 2011; Goldberg & Perry-Jenkins, 2007; Patterson, 1995).

Regarding paid labour, SSC are more likely to be dual-earner couples compared to DSC (Perlesz et al., 2010), hence report a more equal division of paid labour (Jaspers & Verbakel, 2013). Nonetheless, there is also evidence that women in SSC may shift from dual-earners to a single-breadwinner couple after formalizing their union via marriage/civil union, reflecting patters observed among women in DSC (Dillender, 2015).

Looking at research conducted in Italy, in 2001 Barbagli and Colombo published the first study on the Italian LGB population.^4^ Findings from their qualitative interviews suggest that partners in SSC negotiate how to divide housework and, generally, adopt a more egalitarian allocation compared to DSC. Partners who reported an unequal division of unpaid labour also reported either an unequal division of paid labour (e.g., one partner is employed, the other is studying) or, among lesbian SSC, one partner was previously married (with a different-sex partner) and used to be the main responsible for housework chores, hence probably replicating this arrangement also in her SSC.

### Secondary data for studying same-sex couples in Italy

Research on same-sex couples in Italy is hampered by the fact that official statistics do not collect information on sexual orientation and gender identity. The most comprehensive survey on families in Italy, from whose questionnaire we gathered most of our question wording, is the ‘Families, Social Subjects, and Life Cycle’ survey administered by the Italian Institute of Statistics (ISTAT). The last available wave was collected in 2016. Such data source does not allow to identify same-sex couples as information on respondents’ sexual identity is not collected, nor is gender of respondents and their partners: all couples in the sample are coded as different-sex.

On the other hand, existing publicly-available social surveys representative of the Italian population yield too small sample sizes for studying SSC. In Table 1 we describe some of the most commonly used databases to study couples’ dynamics, detailing whether they enable the identification of SSC and, if so, the related sample size. We also include other data sources which are not explicitly targeted to measure the division of labour among partners nor family-related behaviours, but provide large-scale datasets on the non-heterosexual population in Italy.

It follows that most of the existing knowledge on SSC in Italy comes from qualitative studies or ad-hoc (non-representative) surveys (e.g., Baiocco et al., 2014; Barbagli and Colombo, 2007; Castiglioni et al., 2025; Lelleri et al., 2008; Monaco & Nothdurfter, 2023; Saraceno & Bertone, 2003). We contribute to this growing body of literature on the experiences of SSC in Italy.

**Table 1:** Description of existing secondary data for studying same-sex couples and/or division of labour in Italy

Data source	Years	Sample characteristics	Survey aim	*n* SSC	*n* SSC with children
International Social Survey Programme	2018 2019 2020	Respondents aged 18 or older living in Italy	*Annual surveys on diverse topics* *relevant to social sciences* Information on average time spent on domestic labour	Impossible to identify same-sex couples (no gender of partner)	
Fundamental Rights Agency	2023 (Survey III)	Respondents aged 15 or older, living in Italy and identifying as LGBTQIA*	Various topics on the experiences of LGBTQIA* people No information on division of domestic labour	3881 SSC (1588 women and 2293 men in SSC)	5.85% has children (n = 194)
European Social Survey	2016 (Wave 8) 2018 (Wave 9) 2020 (Wave 10)	Respondents aged 18 or older living in Italy	*Public attitudes, beliefs and Behaviours Across Europe* Division of labour for Italy: only in Wave 2—2004 No info on tie with children	n = 420 (163 women and 257 men in SSC). Pooled 2016–2020	18.8% has children (n = 79, 25 women and 54 men in SSC). Pooled 2016–2020
ISTAT UNAR	2021	Italian residents in civil union	*Labour discrimination against LGBT* + *people and diversity policies in enterprises* No information on division of labour	n = 20,189 respondents in civil union or formerly in civil union	n = 1575 respondents in civil union or formerly in civil union
Famiglie e Soggetti Sociali	2016	Respondents aged 18 + living in Italy	*Family dynamics* Information on division of domestic labour and childcare	Impossible to identify same-sex couples (SSC are authomatically recoded as DSC)	
European Union Statistics on Income and Living Conditions	2005–2022	Household members 16 + living in Italy	*Income, poverty, social exclusion and living conditions in EU* No information on division of domestic labour	Impossible to identify same-sex couples (no gender of partner)	

## Data and methods

We collected data on respondents who, at the time of survey, were co-residing with a same-sex partner through an original online survey distributed via mailing lists and websites of Italian associations concerned with LGBTQ* individuals.^5^ The study was conducted in compliance with ethical standards and was approved by the University of Trento Ethics Committee on 29/06/2023 (Prot.2023-033). A pilot of the survey was tested multiple times by the research team before the public distribution. Data collection, designed to be entirely anonymous, took place between 30/06/2023 and 30/07/2023 and resulted in 251 respondents. Among these, 45 did not provide information on key variables of interest, 13 were excluded as they were not in a *co-residential* relationship, 3 were not in a same-sex couple. The remaining 190 respondents (138 women and 52 men in SSC) provided completed responses and were included in the analysis.

We distinguish between couples composed by two women or two men. Although we collected information also on other couple compositions, i.e., man-woman trans* (*n* = 2) and *other* (*n* = 2), we exclude them for the analysis given the small number of observations.

Division of domestic labour was measured using a set of 8 items (grocery shopping, cooking, cleaning, ironing, paying bills, maintenance work, laundry, organising common social activities). The respondents were asked to indicate who, in their household, does each of the eight domestic chores, choosing from 6 answer options: *always me*, *usually me*, *me and my partner equally share this task, usually my partner*, *always my partner*, *usually someone else/not applicable*. Question wording and answer options were taken from ISTAT’s *Families, Social Subjects and Life Cycle* survey,^6^ to provide continuity with existing research on the general population.

We created categorical variables taking value 1 if the respondent always or usually does more of a given task, 2 if the partner always or usually does more, and 3 if the task is shared equally. This latter category encompasses cases where the task was outsourced (for a categorization which excludes such cases, see Table 11 in Appendix). For some descriptive analyses we summarize these eight items into a binary variable (equal vs. unequal division): first we create a mean scale by averaging the scores obtained in each of the 8 items (taking into account the original response scale from 1, always respondent, to 6, always partner); then, we create a dummy variable taking value 0 as unequal division of labour—for mean scores ranging between 1 and 2.375 (always/usually respondent) and between 4.625 and 6 (usually/always partner)—and 1 as equal division—with mean scores ranging between 2.5 and 4.5 (both equally, outsourced/not applicable). These cut-off points were chosen considering that scores close to 1–2 or 5–6 indicate specialization—one partner does substantially more housework than the other –, while scores close to 3 and 4 indicate a more equal division or outsourcing of tasks. This operationalisation is more robust to measurement errors (e.g., if one response is miscoded/unusual it has less impact on the final variable), it does a better job in balancing across tasks (e.g., a respondents that always engages in four tasks and never in the remaining four would be considered as equally sharing), and it is easier to interpret for descriptive analysis compared to other operationalisations.

In order to measure partners’ division of paid work, we consider the working hours of partners and their employment status. For working hours, we indicate whether the respondents and their partners work the same number of hours or not (equal/unequal division of paid labour), and who between the partners works more hours (more respondent, equal hours, more partner). For employment status, we report the presence of asymmetries within the couples (both partners employed, one partner employed, neither partner employed). *Both partners* employed refers to couples with both partners working full- or part-time; *neither partner employed* to couples with both partners inactive, student, retired, unemployed/looking for first job, staying at home, or in another (not specified) status.

Childcare tasks for children aged 0–2 (bathing, changing nappies, going to doctor appointments, getting up at night, staying at home if the child is sick) and for children aged 3–12 (playtime, small outings, bringing/picking up child from school and activities, talking to the school if there are any problems) are used with their original scales (*Mostly me*, *I deal with it more than my partner*, *We both deal with it*, *My partner deals with it more than I do*, *Mostly my partner*, *Not applicable*) and in three categories indicating which partner performs more childcare (More the respondent, More the partner, Both equally) in the cross-tabulations. Childcare questions and answer options were adapted from the Longitudinal Internet studies for the Social Sciences,^7^ a panel largely used for the study of partners’ division of labour (e.g., Yerkes et al., 2020). We distinguish between *birth/biological parents* – those who have a genetic tie to the child, carried the pregnancy, or are parents of a child born or adopted from a previous different-sex relationship – and *social parents* – those whose partner has a genetic tie to the child, whose partner is the birth parent, or whose partner’s child was born or adopted from a previous relationship of the partner. 

### Analytical strategy

Given the non-representative nature of the sample, the small number of observations and the exploratory aim of the research, inferential analysis is not suitable. We therefore employ a descriptive analytical strategy.

## Results

Out of the 190 valid observations, 72.63% (*n* = 138) are from women in SSC. As shown in Table 2, the sample is relatively young and highly educated: 50.53% of respondents are aged 30–39 and 75.79% have at least a bachelor’s degree. Moreover, the couples in our sample are for the majority in a civil union (67.37%) and in long-term relationships: 58.42% have been co-residing for at least 7 years. Overall, the sample composition aligns with findings from previous studies for Italy and other European countries (Van der Vleuten et al., 2021; Vitali et al., 2025). Because the survey was distributed also via LGBTQ* associations that are concerned with same-sex parenthood, a large number of SSC in the sample report having at least one co-resident child. This is especially notable among women in SSC, as less than 20% are childless, compared to roughly 52% of men in SSC.

**Table 2:** Description of the sample by couple composition (*n* = 190)

		Women in SSC (*n* = 138)		Men in SSC (*n* = 52)		Total (*n* = 190)	
		%	n	%	n	%	n
Age of respondent	18–29	4.35	6	3.58	2	4.21	8
	30–39	54.35	75	40.38	21	50.53	96
	40–49	32.61	45	32.69	17	32.63	62
	50 and more	8.79	12	23.08	12	12.63	24
Age of partner	18–29	0.72	1	5.77	3	2.11	4
	30–39	52.17	72	34.62	18	47.37	90
	40–49	39.86	55	38.46		39.47	75
	50 and more	7.25	10	21.15	11	11.05	21
Marital status	Cohabiting	19.75	27	28.85	15	22.11	42
	Civil Union	70.29	97	59.62	31	67.37	128
	Married	10.14	14	11.54	6	10.53	20
Duration of cohabitation	Less than 1 year	2.17	3	1.92	1	2.11	4
	1–3 years	10.87	15	11.54	6	11.05	21
	4–7 years	30.43	42	23.08	12	28.42	54
	More than 7 years	56.52	78	63.46	33	58.42	111
Area of residence	Northern Italy	76.09	105	71.15	37	74.74	142
	Central Italy	18.84	26	17.31	9	18.42	35
	Southern Italy and Islands	5.07	7	3.85	2	4.74	9
	Abroad	0	0	7.69	4	2.11	4
Highest level of education	Middle school	2.90	4	1.92	1	2.63	5
	High school	20.29	28	25.0	13	21.58	41
	Bachelor’s degree or higher	76.81	106	73.08	38	75.79	144
Employment status	Full-time	73.19	101	84.62	44	76.32	145
	Part-time	17.39	24	7.69	4	14.74	28
	Unemployed	0	0	1.92	1	0.53	1
	Stay at home	1.45	2	0	0	1.05	2
	Inactive	2.17	3	1.92	1	2.11	4
	Other	5.80	8	3.85	2	5.26	10
Number of children	0	19.57	27	51.92	27	28.42	54
	1	63.04	87	30.77	16	54.21	103
	2	14.49	20	15.38	8	14.74	28
	3 or more	2.89	4	1.92	1	2.64	5
Age of children*	0–2	47.14	66	42.86	15	46.29	81
	3–5	34.28	48	20.00	7	31.43	55
	6–12	10.71	15	22.85	8	13.14	23
	13 or more	7.86	11	14.29	5	9.14	16

^*^Total number of observations refers to the total number of children reported in the sample (175 children, 140 of women in SSC and 35 of men in SSC)

### Domestic labour

**Fig. 1:**
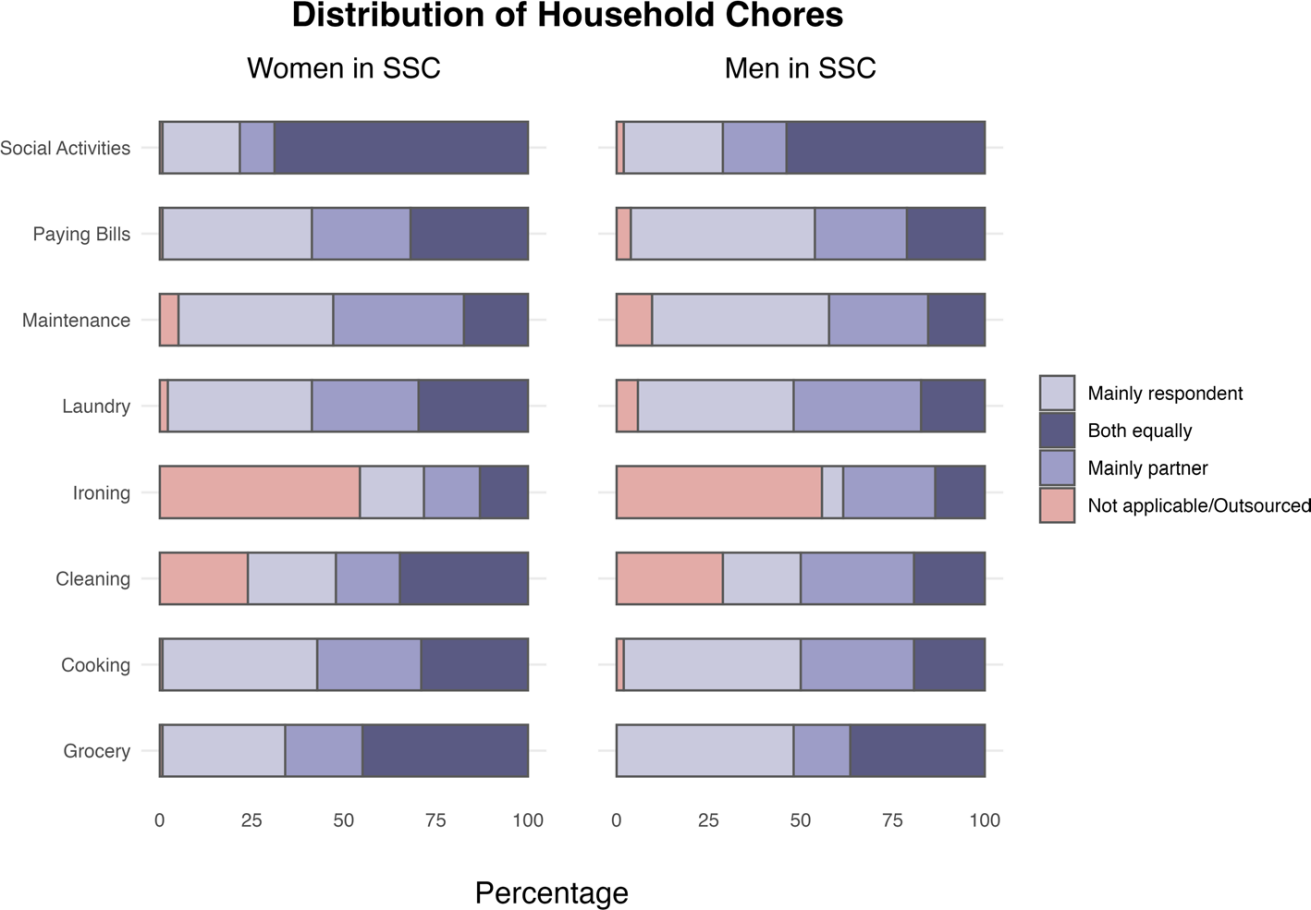
Distribution of domestic chores in same-sex couples, by gender of partners

Overall domestic labour tends to be equally shared by the vast majority (around 90%) of both women and men in SSC (Table 3). This result is robust when considering the presence of children in the household (Appendix, Table 10). When looking at each task separately, we do not find substantial differences between women and men (Fig. 1). Although a non-negligible share of both women and men report outsourcing or not engaging in^8^ ironing and cleaning, for men this pattern expands also to other tasks especially *small maintenance*, *doing the laundry*, *cooking* and *organising social activities*. We performed also additional analysis excluding respondents who outsourced even a single task (Appendix, Table 11)—in Table 3 is outsourcing is considered equal division of tasks—confirming the robustness of our main findings.

**Table 3:** Division of domestic labour in same-sex couples, by gender of partners. Absolute numbers in parentheses

	Division of domestic labour		
	Unequal	Equal	Total
Women in SSC	7.25% (10)	92.75% (128)	100% (138)
Men in SSC	11.54% (6)	88.46% (46)	100% (52)
Total	8.42% (16)	91.58% (174)	100% (190)

While equal division of domestic labour is more prevalent among educationally homogamous couples (95%) compared to heterogamous ones (85%), the vast majority of couples in both groups report sharing domestic tasks equally (Table 4).

**Table 4:** Level of education and division of domestic labour for women and men in SSC

	Division of domestic labour		
	Equal	Unequal	Total
Educational heterogamy	85.3% (58)	14.7% (10)	100% (68)
Educational homogamy	95.1% (116)	4.9% (6)	100% (122)
Total	91.6% (174)	8.4% (16)	100% (190)

### Paid labour

Table 5 reports the share of same-sex couples with an equal vs. unequal division of work hours. About 23% of women in SSC and 25% of men report an exactly equal number of work hours.

**Table 5:** Division of working hours in same-sex couples, by gender of partners. Absolute numbers in parentheses

	Division of working hours		
	Unequal working hours	Equal working hours	Total
Women in SSC	77.54% (107)	22.46% (31)	100% (138)
Men in SSC	75.0% (39)	25.0% (13)	100% (52)
Total	76.84% (146)	23.16% (44)	100% (190)

Partners’ relative employment is reported in Table 6. For both women and men in SSC, both partners are employed—either full- or part-time—in the vast majority of cases (over 80%). Asymmetric employment statuses—where one partner is employed and the other is not– appear to be slightly, more common for women (17%) in SSC than men (12%).

**Table 6:** Employment status in same-sex couples, by gender of partners. Absolute numbers in parentheses. *Not employed* refers to individuals who are unemployed, stay-at-home, retired, inactive or students

	Employment status of couples			
	Both partners employed	Only one partner employed	Neither partner employed	Total
Women in SSC	81.88% (113)	16.67% (23)	1.45% (2)	100% (138)
Men in SSC	86.54% (45)	11.54% (6)	1.92% (1)	100% (52)
Total	83.16% (158)	15.26% (29)	1.58% (3)	100% (190)

Table 7 reports the distribution of domestic labour conditional on the partners’ hours worked. Even when working hours are unequally shared, domestic tasks are still divided equally in most cases. Interestingly, this pattern remains consistent when partners’ relative incomes or employment status are considered (Appendix, Tables 12 and 13, respectively).

**Table 7:** Division of working hours and domestic labour in same-sex couples. Absolute numbers in parentheses

Hours worked	Division of domestic labour			Total
	More respondent	Equal	More partner	
More respondent	7.35% (5)	91.18% (62)	1.47% (1)	100% (68)
Equal	4.55% (2)	93.18% (41)	2.27% (1)	100% (44)
More partner	7.69% (6)	91.03% (71)	1.28% (1)	100% (78)
Total	5.79% (13)	91.05% (173)	3.16% (3)	100% (190)

### Childcare

**Fig. 2:**
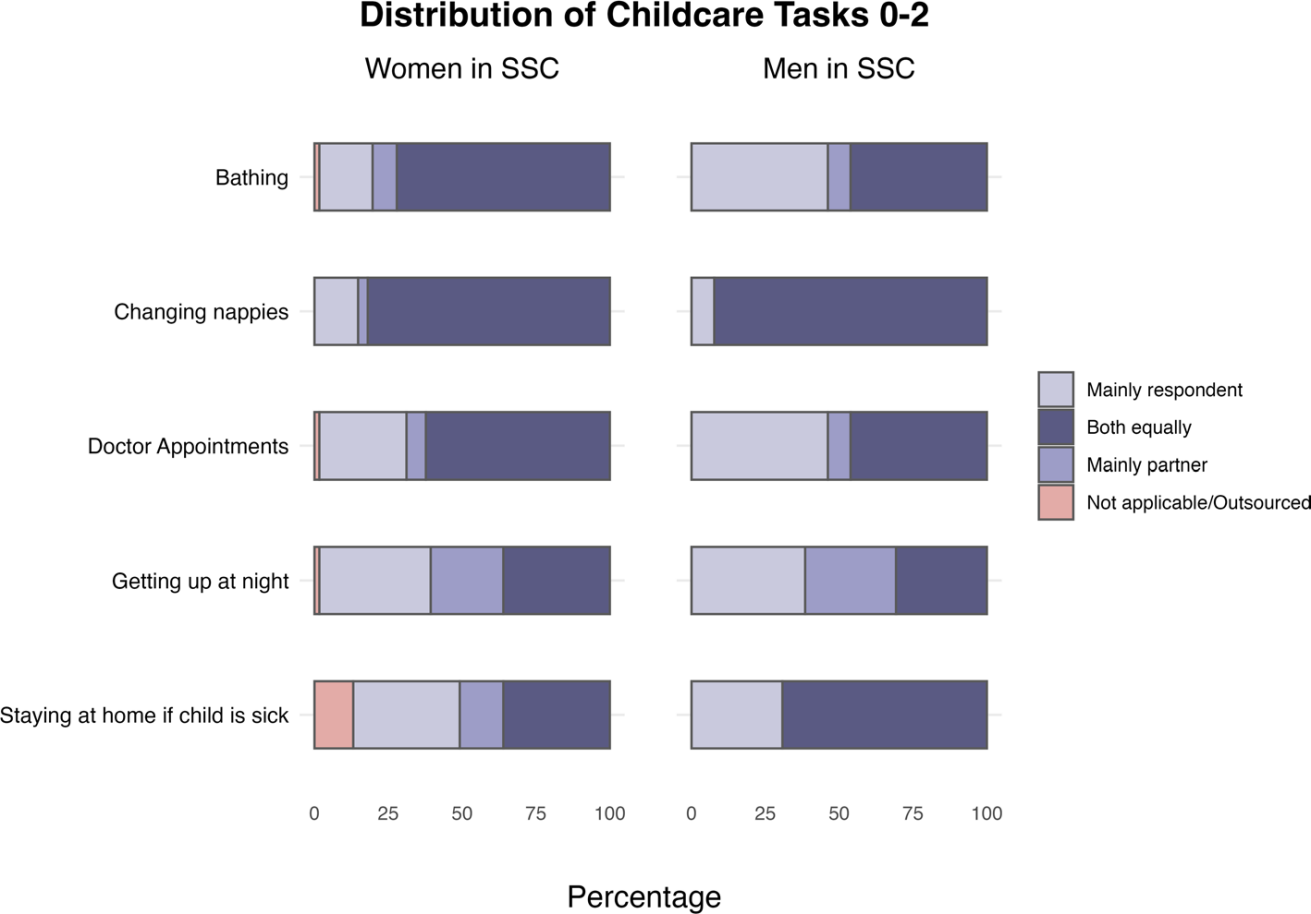
Distribution of childcare tasks in same-sex couples for children aged 0–2, by gender of partners

**Fig. 3:**
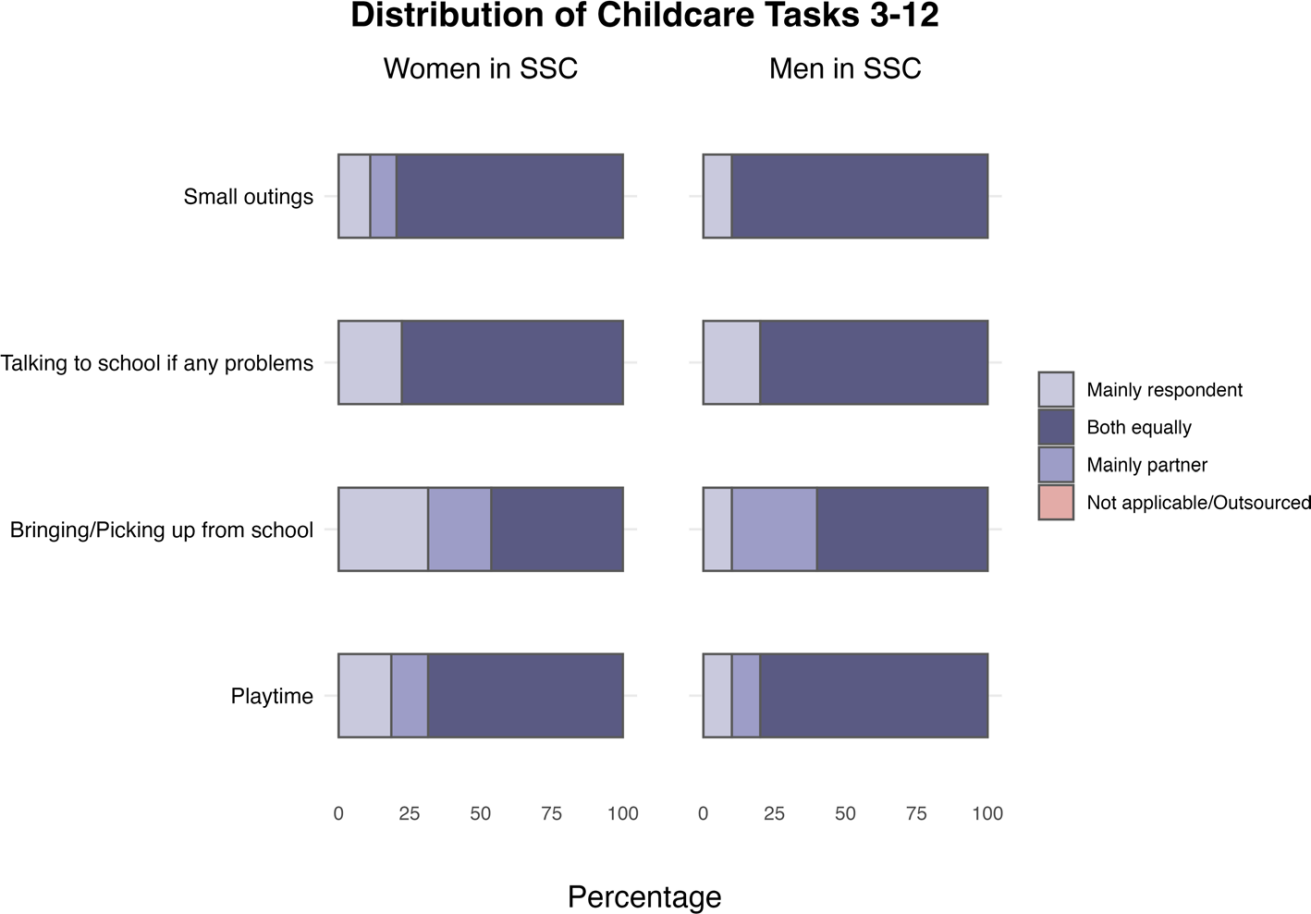
Distribution of childcare tasks in same-sex couples for children aged 3–12, by gender of partners

Figures 2 and 3 report same-sex parents’ involvement in each childcare task by age of the youngest child. Overall, there are no particular differences between SSC of women and men in terms of childcare towards children aged 0–2 and 3–12. An exception applies, limited to children aged 0–2, concerning tasks such as *Bathing* and *taking the child to doctor appointments:* women in SSC tend to engage in these tasks more equally than men in SSC do. Men, instead, report more equality in *staying at home when child is sick* than women do.

**Fig. 4:**
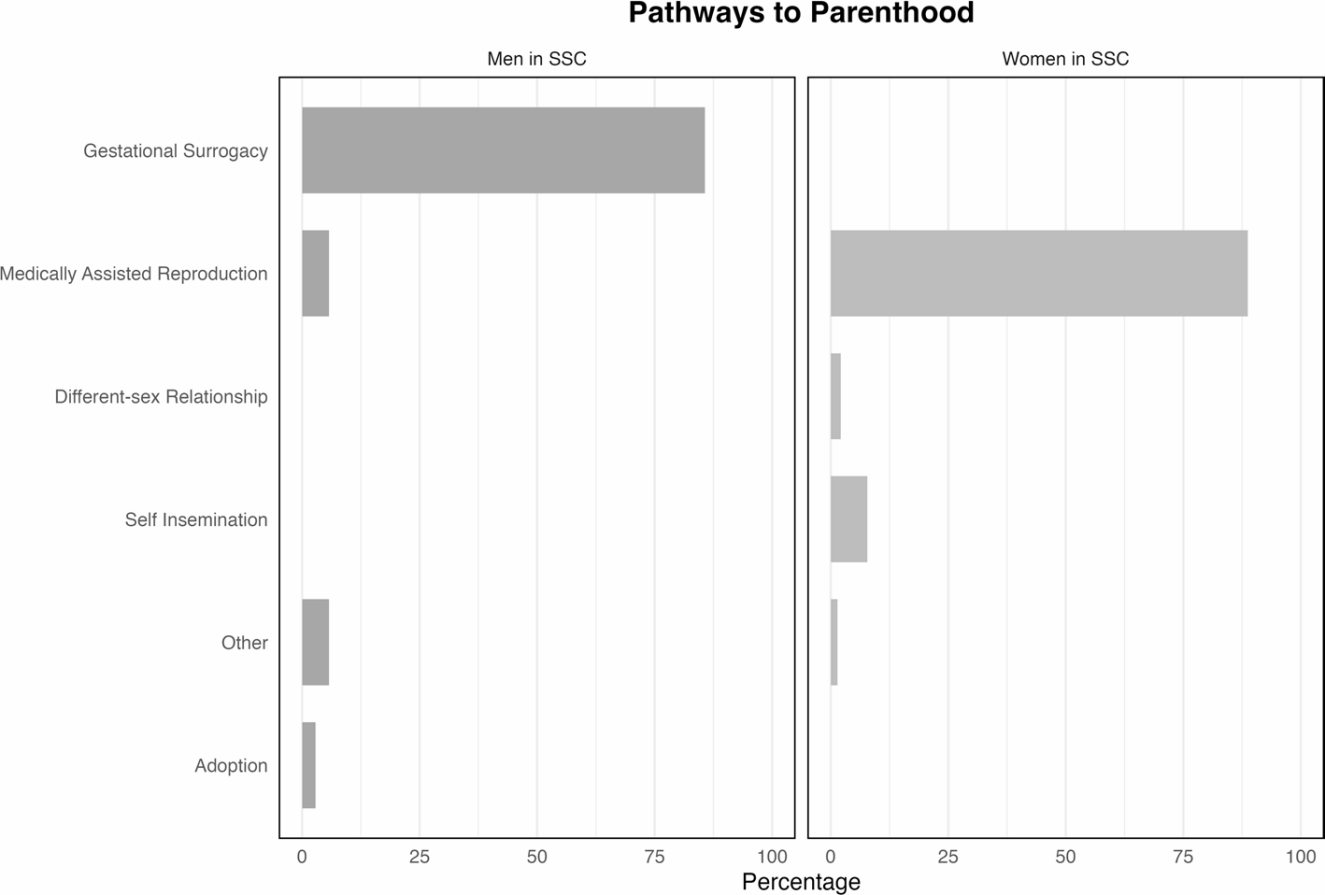
Pathways to parenthood for each child among same-sex couples, by gender of partners (number of children = 150)

Tables 8 and 9 summarize the distribution of childcare by age of child into one indicator. Parents are considered birth/biological when they have a genetic tie with the child, carried the pregnancy, or the child was born from their previous heterosexual relationship.^9^ We considered situations where two or more children of the same couple have different ties with their parents (e.g., the respondent is the social parent for one child and biological/birth parent another). In most of these cases, the children had different ages and, therefore, different sets of questions regarding childcare tasks. This allowed us to create two *parent tie* variables, one for measuring ties with children aged 0–2 years (*n* = 71) and one for those aged 3–12 years (*n* = 62) (Appendix, Fig. 4).

**Table 8:** Division of childcare tasks of children aged 0–2 between partners for women (*n* = 50) and men (*n* = 11) in same-sex couples. Absolute numbers in parentheses

Respondent’s tie with children	Division of childcare 0–2							
	Women in SSC				Men in SSC			
	More respondent	Both equally	More partner	Total	More respondent	Both equally	More partner	Total
Birth/Biological Parent	28.12% (9)	71.88% (23)	0	100% (32)	33.33% (2)	66.67% (4)	0	100% (6)
Social Parent	5.56% (1)	88.89% (16)	5.56% (1)	100% (18)	20% (1)	80% (4)	0	100% (5)
Total	20% (10)	78% (39)	2% (1)	100% (50)	27.27% (3)	72.73% (8)	0	100% (11)

Childcare is calculated as summary measure of five childcare tasks (Bathing, Changing nappies, Going to doctor appointments, Getting up at night, Staying at home if the child is sick)

**Table 9:** Division of childcare tasks of children aged 3–12 between partners for women (*n* = 52) and men (*n* = 8) in same-sex couples. Absolute numbers in parentheses

Respondent’s tie with children	Division of childcare 3–12							
	Women in SSC				Men in SSC			
	More respondent	Both equally	More partner	Total	More respondent	Both equally	More partner	Total
Birth/Biological Parent	12.50% (3)	83.33% (20)	4.17% (1)	100% (24)	0	100% (4)	0	100% (4)
Social Parent	3.57% (1)	82.14% (23)	14.29% (4)	100% (28)	0	100% (4)	0	100% (4)
Total	7.69% (4)	82.69% (43)	9.62% (5)	100% (52)	0	100% (8)	0	100% (8)

Childcare is calculated as summary measure of four childcare tasks (Playtime, Small Outings, Bringing/Picking up child from school and activities, Talking to the school if there are any problems)

Adoption is not allowed for same-sex couples in Italy. We nonetheless included in the survey answer options for adoption and fostering because the social parent can be legally recognized^10^ as a step-parent, regardless of the genetic tie with the child, via a process referred to as ‘stepchild adoption’.

Results in Tables 8 and 9 show that, in our sample, childcare tasks are, on average, equally divided between partners in the majority of cases across both men and women in SSC.

Yet, when the respondents are the birth/biological parents, they tend to over-report being the main provider of care for young children, in both SSC of women and men (Table 8). There is therefore some evidence of specialisation. Parents’ involvement in childcare become more equal for older children (Table 9). In our sample of eight men in SCC with children aged 3–12, all of them share childcare equally, independently of their tie with the child(ren). Among women in SSC, no particular difference in reporting emerges between biological vs. social mothers.

## Discussion

This contribution provides a first description of the division of paid and unpaid labour among same-sex couples in Italy on the basis of non-representative data collected through an online survey. Although the sample is not representative, and it only comprises 190 cases, it serves the purpose of contributing to a growing body of literature on the LGBTQI* population in Italy, by describing understudied patterns of paid and unpaid labour among same-sex couples which could not otherwise be studied due to lack of representative data or larger samples.

In line with the existing literature on other countries, we found an overall equal distribution of paid and unpaid labour among same-sex partners in our sample. Results suggest that family practices in same-sex couples diverge from the predominant conservative division of domestic labour found in the general population in Italy. Not only both women and men in same-sex couples tend to equally divide domestic labour, but they do it regardless of the presence of children—something that instead deeply impacts the distribution of housework in different-sex couples (Anxo et al., 2011; Mencarini & Tanturri, 2004; Zannella & De Rose, 2020). For instance, in our sample, 30% of SSC where both partners are employed divide equally preparing meals, 47% grocery shopping and 35% cleaning. According to ISTAT’s 2016 data, the share is lower among DSC with the same characteristics, equal to around 20% for cooking, 36% for grocery shopping and 21% for cleaning.^11^ Outsourcing of tasks such as cleaning, which in our sample is around 27% for employed SSC, in the case of DSC only accounts for less than 4%. When considering the presence of children in the household, only 19% of DSC equally shares a task such as cleaning the house, for instance, while more than 33% in our sample of SSC do so.

The tendency to divide equally domestic labour also applies when same-sex partners have an unequal division of paid work: even in couples where one partner works more hours or earns more than the other, domestic labour tends to be equally shared, differently than for different-sex couples. For instance, only about 55% of the DSC with unequal working hours equally share domestic labour^10^, while it is 70% in the case of our sample of SSC.

Moreover, in our sample, domestic tasks are equally shared even when there is a difference in partners’ relative income. This consistency in egalitarian practices among both women and men in same-sex couples contrasts with the gendered division of roles in different-sex couples. Obviously, ISTAT’s data on DSC date back to 2016, while our data on SSC was collected in 2023: it is possible that during the past years, behaviours among DSC have become more egalitarian. A new survey on DSC was fielded in 2024: it will be important to monitor its results.

By disrupting the breadwinner/homemaker model, same-sex partners may be actively rejecting conservative gender roles and expectations, resulting in practices that diverge substantially from those seen in different-sex couples. At the same time, we also find that women in same-sex couples may tend to replicate behaviours commonly observed in heterosexual women: women engage in housework and childcare tasks, independently of their and their partners’ employment status, education or income, and of their biological vs. social role. Men in our sample, instead, engage less in unpaid work, particularly housework, and more often rely on outsourcing, thus mirroring patterns typically found for men in different-sex couples. Further research with more comprehensive data is needed to dig deeper into these dynamics.

We acknowledge the possibility that respondents in same-sex couples may over-report egalitarian practices to abide to social norms: independently of gender, individuals in SSC may be expected to share tasks equally. To eliminate eventual social-desirability bias, future research should measure the division of labour among SSC relying on time diaries or compare self-reported answers provided to questionnaires collected from both partners.

Similarly, whether looking at the amount of hours that partners spend in the labour market or at the employment status of the couple, we do not find any substantial difference in the distribution of paid labour between women and men in same-sex couples: in the vast majority of SSC, both partners are employed. These results are in line with existing literature on SSC in other high-income countries (Jaspers & Verbakel, 2013; Perlesz et al., 2010).

Lastly, in those (few) couples where one partner is more involved than the other in childcare, we find only partial proof of specialisation. Interestingly, we do not see a strong specialised distribution of childcare. A slight pattern does emerge among women with younger children (aged 0–2), but it is less pronounced than expected. We expected an even stronger involvement of the birth/biological mother considered the physiological involvement and the difficulties in accessing nurseries in the Italian context, for instance.

Our study presents several limitations. First, the small size and non-representative nature of our sample. While many socio-economic characteristics of our sample align with representative data on Italian same-sex couples^12^ (e.g., Vitali et al., 2025), further research with probabilistic and larger samples is needed to enhance the robustness of these findings and go beyond a purely descriptive analysis. Second, the associations used to recruit participants via mailing list oversampled respondents with co-residing children. Indeed, many of the associations involved provide support to prospective same-sex parents with navigating cross-border medically assisted reproduction, step-child adoption practises and same-sex parenthood-related support. Third, the small number of childless couples does not allow for an in-depth comparison of the division of labour between same-sex couples with and without children. Furthermore, the small number of sampled men, prevented us from better unfolding gender differences. All in all, the scarce variability of the sample composition especially for what concerns education and presence of children is a limit of our study.

Moreover, applying heteronormative models to explain the experiences of partners of the same gender presents several shortcomings, such as disregarding the impact of societal marginalisation and overlooking the unique power dynamics typical of these relationships (Kelly & Hauck, 2015).

Finally, due to restrictions imposed by the Ethics Committee, it was not possible to directly ask about the sexual identity of respondents, as this information is classified as sensitive data and was considered as beyond the scope of this study. This limitation excludes bisexual identities. Further research should consider sexual identity to better understand variations within the LGBTQ* population.

Despite these limitations, we provide descriptive evidence on how women and men in same-sex couples divide paid and unpaid labour in Italy. Same-sex couples are becoming increasingly more widespread in Italy: since the second half of 2016—when they become available—to the end of 2023, over 21,000 civil unions between same-sex partners were celebrated and supposedly many more are co-residing and difficult to be captured by official statistics. More research is needed to describe and understand how partners in same-sex couples divide paid and unpaid labour in Italy. More generally, sociodemographic research should devote systematic attention to same-sex families, particularly in Italy, where attitudes are becoming more favourable towards the LGBTQ* population and LGBTQ* families– 47% of Italians considered homosexuality unjustifiable in the period 2005–2009, while only 15% think so in 2017–2022—but remain still significantly behind compared to other European countries (Vitali et al., 2025). Despite some legal advancements in the recognition of same-sex families—often achieved to court rulings rather than legislative reforms—Italy continues to lack comprehensive legal protection and recognition of these families (Vitali et al., 2025). Recent developments paint an even more troubling scenario: a rise in hate crimes based on sexual and gender identity,^13^ and the criminalisation of gestational surrogacy, including when it occurs abroad,^14^ reflect an increasingly hostile climate for the LGBTQ* population.

## Data Availability

The data collected for the current study is not publicly available due to privacy restrictions imposed by the Ethics Committee of the University of Trento.

## Appendix

Figure 4 and Tables 10, 11, 12 and 13.

**Table 10:** Presence of children and division of domestic labour in same-sex couples, by gender of partners

Division of domestic labour	Women in SSC			Men in SSC		
	Unequal	Equal	Total	Unequal	Equal	Total
No children	11.11% (3)	88.89% (24)	100% (27)	11.11% (3)	88.89% (24)	100% (27)
At least one child	6.31% (7)	93.69% (104)	100% (111)	12.00% (3)	88.00% (22)	100% (25)
Total	7.25% (10)	92.75% (128)	100% (138)	11.54% (6)	88.46% (46)	100% (52)

**Table 11:** Division of domestic labour between in same-sex couples, by gender of partners. Outsourcing is excluded from the analysis

	Division of domestic labour		
	Unequal	Equal	Total
Women in SSC	18.18% (10)	81.82% (45)	100% (55)
Men in SSC	30.00% (6)	70.00% (14)	100% (20)
Total	21.23% (16)	78.67% (59)	100% (75)

**Table 12:** Division of domestic labour and partners’ contribution to the total couple’s incomes in same-sex couples, by gender of partners. Sample size differs from the main analysis because of missing values in the variable *partners’ contribution to the total couple’s incomes*

Partners’ contribution to the total couple’s incomes	Division of domestic labour			
	More respondent	Equal contribution	More partner	Total
More respondent	4.00% (2)	96.00% (48)	0	100% (50)
Equal contribution	3.95% (3)	94.74% (72)	1.32% (1)	100% (76)
More partner	9.38% (3)	87.50% (28)	3.12% (1)	100% (32)
Total	5.06% (8)	93.67% (148)	1.27% (2)	100% (158)

**Table 13:** Employment status of the couple and division of domestic labour

Employment status couple	Division of domestic labour		
	Equal	Unequal	Total
Both partners employed	93.67% (148)	6.33% (10)	100% (158)
Only one partner employed	82.76% (24)	17.24% (5)	100% (29)
Neither partner employed	66.67% (2)	33.33% (1)	100% (3)
Total	91.58% (174)	8.42% (16)	100% (190)

## Footnotes

1 Source: ISTAT, Resident population on 1st January 2024. Available at: https://demo.istat.it/app/?i=POS&a=2024&l=en.

2 Source: ISTAT (2016). Report: I Tempi della Vita Quotidiana Anno 2014. https://www.istat.it/wp-content/uploads/2016/11/Report_Tempidivita_2014.pdf (last visited 23/07/2025).

3 In Italy, school is mandatory starting from age 6. However, above 90% of all children in the Italian territory attend kindergarten (3–5 years old). Instead, the shares of children attending nursery school (0–2 years old) are much lower (30.7%). Source: ISTAT (2025). Report “I servizi educativi per l’infanzia in Italia”. https://www.istat.it/wp-content/uploads/2025/05/report_infanzia_2023_2024.pdf (last visited: 22/07/2025).

4 In this paper we refer to the second edition of the book, Barbagli and Colombo (2007)

5 Namely Agedo, Arcigay, Famiglie Arcobaleno, Osservatorio LGBT Università Federico II Napoli, Rete Genitori Rainbow.

6 Questionnaire available at https://www.istat.it/microdati/multiscopo-sulle-famiglie-famiglie-soggetti-sociali-e-ciclo-di-vita/ (Accessed 1st December 2024).

7 https://www.lissdata.nl/ (Accessed 1st December 2024).

8 *Outsourcing* and *not applicable* were a single answer option. We hypothesise that, in most cases, selecting this option reflects outsourcing rather than inapplicability, as it pertains to basic need tasks.

9 In the case of women in SSC who adopted the ROPA method (i.e., Reception of Oocytes from a Partner, a type of in vitro fertilisation, where one of the partners carries on the pregnancy with the embryos of the other partner) for conceiving their child, we consider the birth mother the one who carried the pregnancy, and the social mother the with a genetic tie with the children. This distinction was made in light of previous literature exploring how pregnancy and childbirth can impact partners’ division of labour. We also maintain the difference between birth mother and social mother in one case of two women who procreated with embryo donation.

10 All respondents who selected both the *adoptive parent* option and the *other* option specified, in the free text provided, that they were legally granted stepchild adoption.

11 Source: own elaborations from publicly available ISTAT’s data “Famiglie, soggetti social e ciclo di vita” (Families, Social Subjects, and Life Cycle) (2016). Sample of 4882 respondents in different-sex cohabiting couples (2492 women and 2390 men), aged 18–64, both partners employed. Weighted.

12 Source: ISTAT, survey on Civil Partnerships, available at: https://demo.istat.it/tavole/?t=unionicivili&l=en.

13 ILGA-Europe report of 2025 available at: https://www.ilga-europe.org/files/uploads/2025/02/ILGA-Europe-Annual-Review-2025-Bias-Motivated-Violence.pdf (last visited 25/07/2025).

14 Law No. 169 of November 4, 2024. (https://www.gazzettaufficiale.it/eli/id/2024/11/18/24G00187/sg).
